# Keystone Veterinary Medical Association

**Published:** 1895-10

**Authors:** W. L. Rhoads

**Affiliations:** Secretary


### KEYSTONE VETERINARY MEDICAL ASSOCIATION.

THE September meeting was called to order by President Lintz at the office of Dr. W. H. Hoskins, 3452 Ludlow Street, Philadelphia, Tuesday evening, September 17, 1895, when Drs. W. H. Hoskins, J. R. Hart, Charles Lintz, W. L. Rhoads, and J. T. McAnulty answered to roll-call. A communication from Dr. Bridge was read expressing his regret in being unable to attend, as he had been called to Bobesonia.

The chairman of the Legislative Committee reported very little excitement in that line since our last meeting, as there had not been any appointments made on the State Board of Veterinary Medical Examiners or for State Veterinarian.

At the request of the Treasurer and Secretary, President Lintz appointed as a committee to audit the books Drs. J. R. Hart and W. H. Hoskins.

Dr. J. R. Hart, delegate to the meeting of the Pennsylvania State Veterinary Medical Association, at Cresson, gave a very interesting account of that meeting, which proved very entertaining and instructive, though small, as those veterinarians who wanted it there forgot to attend.

Dr. Hoskins, who was the delegate to the meeting of the United States Veterinary Medical Association, at Des Moines, Iowa, stated that it was the largest convention of veterinarians ever held. He gave a glowing account of their reception and entertainment offered at Chicago. This, with his full though concise report of the convention, banquet, etc., made the mouth of the poor stay-at-home water practically as well as figuratively speaking, and their watchword for next year is "On to Buffalo or bust."

Adjourned to meet October 8th at the northwest corner of Broad and Filbert Streets, in their new quarters. Officers for the ensuing year will be elected.

W. L. RHOADS,
Secretary.

[1] See page 649. [3] See page 640. [2 4 5] Will be published next month.

